# Machine learning approaches to predict peak demand days of cardiovascular admissions considering environmental exposure

**DOI:** 10.1186/s12911-020-1101-8

**Published:** 2020-05-01

**Authors:** Hang Qiu, Lin Luo, Ziqi Su, Li Zhou, Liya Wang, Yucheng Chen

**Affiliations:** 1 School of Computer Science and Engineering, University of Electronic Science and Technology of China, No.2006, Xiyuan Ave, West Hi-Tech Zone, 611731 Chengdu, Sichuan P.R. China; 2 Big Data Research Center, University of Electronic Science and Technology of China, Chengdu, China; 3 Department of Statistics, Faculty of Science, University of British Columbia, Vancouver, Canada; 4 Health Information Center of Sichuan Province, Chengdu, China; 5 Cardiology Division, West China Hospital, Sichuan University, Chengdu, China; 6 West China Biomedical Big Data Center, West China Hospital, Sichuan University, Chengdu, China

**Keywords:** Machine learning, Cardiovascular disease, Hospital admission, Prediction, Environmental exposure

## Abstract

**Background:**

Accumulating evidence has linked environmental exposure, such as ambient air pollution and meteorological factors, to the development and severity of cardiovascular diseases (CVDs), resulting in increased healthcare demand. Effective prediction of demand for healthcare services, particularly those associated with peak events of CVDs, can be useful in optimizing the allocation of medical resources. However, few studies have attempted to adopt machine learning approaches with excellent predictive abilities to forecast the healthcare demand for CVDs. This study aims to develop and compare several machine learning models in predicting the peak demand days of CVDs admissions using the hospital admissions data, air quality data and meteorological data in Chengdu, China from 2015 to 2017.

**Methods:**

Six machine learning algorithms, including logistic regression (LR), support vector machine (SVM), artificial neural network (ANN), random forest (RF), extreme gradient boosting (XGBoost), and light gradient boosting machine (LightGBM) were applied to build the predictive models with a unique feature set. The area under a receiver operating characteristic curve (AUC), logarithmic loss function, accuracy, sensitivity, specificity, precision, and F1 score were used to evaluate the predictive performances of the six models.

**Results:**

The LightGBM model exhibited the highest AUC (0.940, 95% CI: 0.900–0.980), which was significantly higher than that of LR (0.842, 95% CI: 0.783–0.901), SVM (0.834, 95% CI: 0.774–0.894) and ANN (0.890, 95% CI: 0.836–0.944), but did not differ significantly from that of RF (0.926, 95% CI: 0.879–0.974) and XGBoost (0.930, 95% CI: 0.878–0.982). In addition, the LightGBM has the optimal logarithmic loss function (0.218), accuracy (91.3%), specificity (94.1%), precision (0.695), and F1 score (0.725). Feature importance identification indicated that the contribution rate of meteorological conditions and air pollutants for the prediction was 32 and 43%, respectively.

**Conclusion:**

This study suggests that ensemble learning models, especially the LightGBM model, can be used to effectively predict the peak events of CVDs admissions, and therefore could be a very useful decision-making tool for medical resource management.

## Background

Cardiovascular diseases (CVDs) are the leading cause of death worldwide; about 17.9 million deaths were attributable to CVDs in 2016, representing approximately 31% of all global deaths in that year [1]. Even though behavioral factors, including physical inactivity, smoking, unhealthy diets and obesity, are well-known risk factors for CVDs, a large body of studies have indicated that environmental exposure [2–4], such as ambient air pollution [5–9] and temperature variability [10–12], also makes a significant contribution to CVDs, resulting in increased risk of morbidity. For example, using conditional logistic regression models, Liu et al. [13] conducted a multi-city study in 26 Chinese cities, and the results showed that elevated concentrations of sulfur dioxide (SO_2_), nitrogen dioxide (NO_2_), carbon monoxide (CO), and ozone (O_3_) were associated with increased risk of hospitalization for heart failure. Another national time-series study conducted in 184 Chinese cities linked temperature variability to the increase of hospital admissions for CVDs and its subtypes using over-dispersed Poisson regression models [14]. Although these statistical regression models can assess the associations of environmental exposure with CVDs morbidity [15–17], they are often incapable of providing sufficiently accurate morbidity prediction for healthcare management. Moreover, we lack information on the effect of a complex mixture of environmental exposure on CVDs morbidity.

With an increasing number of CVDs patients putting pressure on the limited medical resources, the prediction of healthcare demands, particularly those associated with peak events, has gained greater attention. Time series forecasting approaches, such as the autoregressive integrated moving average (ARIMA) model and the seasonal ARIMA model, are widely applied in predicting problems regarding emergency department visits [18, 19], new admission inpatients [20] and inpatients discharge [21]. However, these models have difficulties solving the complex nonlinear relationship among multi-factors, and their forecasting abilities to extrapolate are limited.

Recently, machine learning algorithms, which can solve the nonlinear relationship among multi-dimensional variables, have been shown to be effective in prediction, and are being used successfully in various healthcare applications, such as medical diagnosis [22, 23] and disease risk prediction [24, 25]. Nevertheless, only a very limited number of studies have attempted to adopt machine-learning based data-driven approaches to forecast the demand for healthcare services associated with environmental exposure, and these few studies predominately focused on the application of artificial neural network (ANN) [26–29]. For instance, Kassomenos et al. [30] applied ANN and stepwise regression models to predict the daily number of hospital admissions for CVDs and respiratory diseases considering air pollution and meteorological conditions, and ANN performed better than the regression model. Moreover, there were relatively fewer machine-learning based studies on predicting peak event of healthcare demand associated with environmental exposure [31]. To the best of our knowledge, only one study has used ANN to forecast peak demand days of emergency department visits for chronic respiratory diseases based on weather and environmental pollution. Although part of other machine learning algorithms performed better than ANN in other fields [32], it is unclear how effective the other machine learning approaches are in predicting the healthcare services demand associated with environmental exposure, which leaves open the potential for the development of more accurate predictive models using other algorithms.

In this study, we contribute to the existing body of knowledge by developing and comparing various machine learning models in predicting the peak demand days of CVDs admissions based on hospital admissions data, air quality data and meteorological data in Chengdu, China from 2015 to 2017. Six types of machine learning models, including logistic regression (LR), support vector machine (SVM), ANN, random forest (RF), extreme gradient boosting (XGBoost), and light gradient boosting machine (LightGBM), were constructed, and their predictive performances were also compared. The study shows the potential of machine learning approaches for predicting peak events of CVDs admissions, and identifies the most sui model for decision making.

## Methods

### Overview of the research framework

**Fig. 1:**
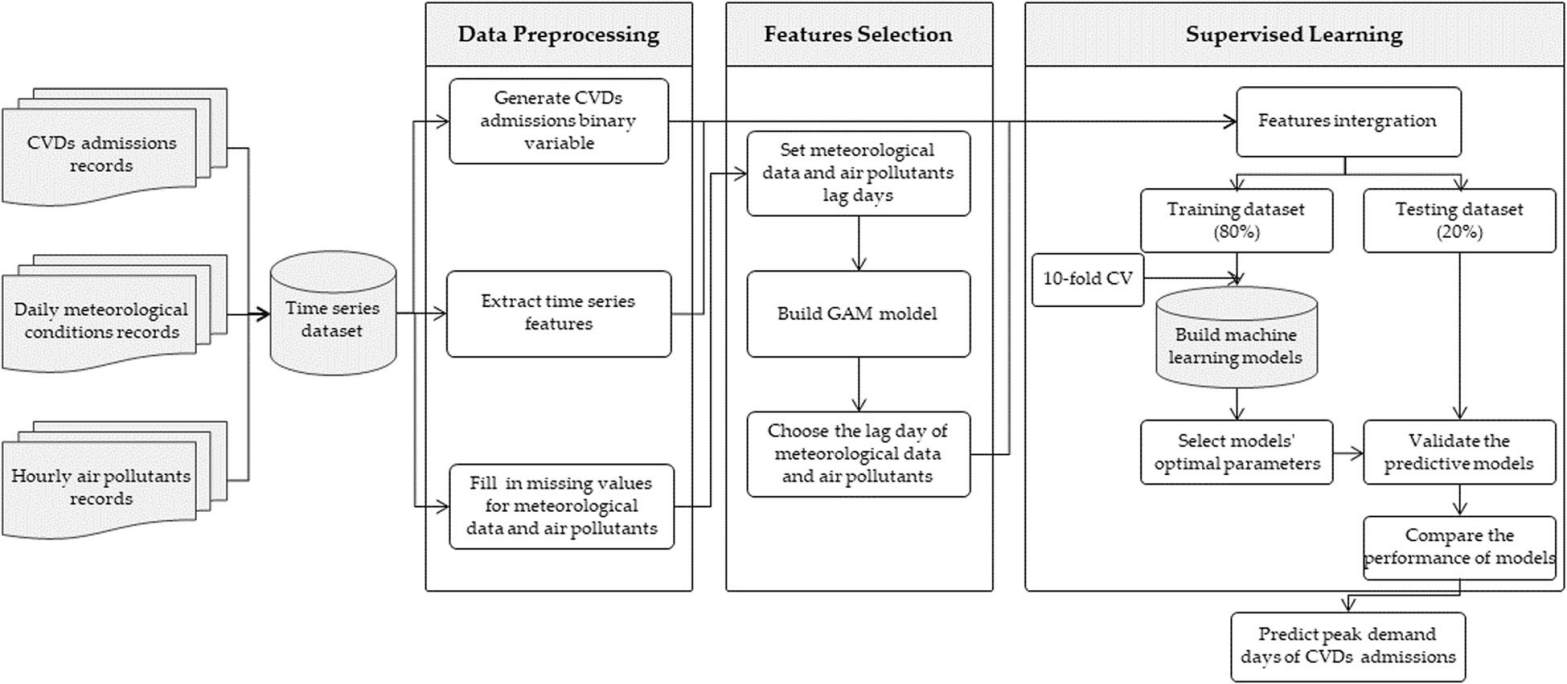
Block Diagram of Classified Prediction Process

This study attempted to predict the peak demand days of CVDs admissions using machine learning techniques. The block diagram of the classified prediction process is shown in Fig. 1. In brief, the time series dataset, which was comprised of CVDs admissions, meteorological data and air quality data, was pre-processed. Second, the generalized additive model (GAM) was built to choose the lag day of meteorological conditions and air pollutants for CVDs admission. Then, six machine learning algorithms, including LR, SVM, ANN, RF, XGBoost and LightGBM, were applied to construct the predictive models, and the models’ parameters were optimized with 10-fold cross validation. After that, the predictive models were validated, then the performances of these models were compared. Finally, we predicted the peak demand days of CVDs admissions based on the optimal machine learning model.

The details are discussed in the following sub-sections.

### Data collection and preprocessing

#### Hospital admissions data

Data for the daily number of hospital admissions for patients with CVDs who lived in urban areas of Chengdu was obtained from the Health Information Center of Sichuan Province, China. This data contains aggregate numbers of CVDs admissions in all the tertiary and secondary hospitals of Chengdu each day with primary diagnosis of CVDs (International Classification of Diseases, 10th Revision codes: I00-I99) from 1 January 2015 to 31 December 2017, which is 1096 days of continuous data.

Additionally, we focused on the peak demand of CVDs admissions, and the binary variable was generated from the daily number of CVDs admissions. In the absence of a known threshold for daily CVDs admissions, the peak demand was defined on the basis of an 85th percentile threshold (304 hospital admissions per day) by reference to the previous studies [31, 33]. Specifically, the days on which the daily number of CVDs admissions were equal to or above the 85th percentile threshold were defined as peak demand days. Thus, the binary variable of CVDs admissions is highly imbalanced, with 931 samples of non-peak demand and 165 samples of peak demand. This binary variable of CVDs admissions was used as the primary dependent variable in the analysis.

#### Meteorological data and air quality data

Meteorological data, including temperature, relative humidity and rainfall, were derived from the Chengdu Meteorological Monitoring Database (http://data.cma.cn/).

Hourly data of air pollutants, including PM_2.5_ (particulate matter with aerodynamic diameter ≤ 2.5 μm), PM_10_ (particulate matter with aerodynamic diameter ≤ 10 μm), SO_2_, NO_2_, CO and O_3_, were obtained from the China National Environmental Monitoring Center (http://www.cnemc.cn/), which provides real-time monitoring of hourly concentrations of air pollutants to the general public. We averaged the 24-h mean concentrations for PM_2.5_, PM_10_, SO_2_, NO_2_ and CO, and calculated maximum 8-h moving average concentrations for O_3_ from the air quality monitoring stations interspersed among the urban areas of Chengdu. Concentrations of particulate matter with an aerodynamic diameter between 2.5 and 10 μm (PM_C_) were calculated by subtracting daily average concentrations of PM_2.5_ from PM_10_ [9, 34].

#### Data preprocessing

Data for the daily number of hospital admissions for CVDs, meteorological data and air quality data were collected from different data sources. We merged these three datasets to form a time series dataset by date (i.e. 1 January 2015 to 31 December 2017). The time series features were extracted from date, including year, month (month of year), day (day of month), holiday (public holidays) and DOW (day of week).

During the study period, the percentages of missing values from the monitoring stations were 1.28% (14/1096) for meteorological conditions, and 3.19% (35/1096) for air pollutants. The linear interpolation which has acceptable performance and reliability was used to fill in the missing values of meteorological conditions and air pollutants [35, 36].

### Feature extraction

As illustrated in the above section, the features for predicting the peak demand days of CVDs admissions included time series features, meteorological condition features and air pollutant features. Accumulating epidemiological studies have suggested that the effect of meteorological conditions and air pollutants on CVDs admissions is delayed, and the lag effect is related to the regional environment [8, 12, 37]. Hence, we employed an over-dispersed GAM, which allowed the quasi-Poisson distribution to analyze the lag effects of daily meteorological conditions and air pollutants on CVDs admissions, and chose the lag day based on the minimum Generalized Cross-Validation (GCV) values which measure models fit [5, 34]. The lag effects of single day lags (from lag0 to lag6) and cumulative day lags (from lag01 to lag06) were taken into consideration. The penalized spline approaches were applied to control for potential confounding of long-term trends, seasonality and meteorological effects [38]. Moreover, dummy variables of holiday and DOW were controlled.

The results demonstrated that temperature, relative humidity, rainfall, PM_2.5_, PM_10_, PM_C_, SO_2_, NO_2_, CO and O_3_ were associated with CVDs admissions, with the minimum GCV values at lag04, lag06, lag06, lag3, lag3, lag3, lag0, lag0, lag0 and lag6, respectively.

Finally, the independent variables for forecasting the peak demand days of CVDs admissions included fifteen features, which are shown in Table 1.

**Table 1:** The features for prediction

**Feature category**	**Features**	**Description**
**Time series features**	year	year of the date of hospital admission
	month	month of year
	day	day of month
	holiday	public holidays
	DOW	day of week
**Meteorological condition features**	Tem_lag04	mean temperature for the moving average of current day and previous four days (lag04)
	RH_lag06	relative humidity for the moving average of current day and previous six days (lag06)
	Rain_lag06	rainfall for the moving average of current day and previous six days (lag06)
**Air pollutants features**	PM2.5_lag3	PM_2.5_ at the previous three days (lag3)
	PM10_lag3	PM_10_ at the previous three days (lag3)
	PMC_lag3	PM_C_ at the previous three days (lag3)
	SO2_lag0	SO_2_ at the current day (lag0)
	NO2_lag0	NO_2_ at the current day (lag0)
	CO_lag0	CO at the current day (lag0)
	O3**_**lag6	O_3_ at the previous six days (lag6)

### Machine learning methods

In this study, six well-accepted machine learning algorithms, including LR, SVM, ANN, RF, XGBoost and LightGBM, were applied to develop predictive models with the unique feature set. These machine learning methods were considered according to their following characteristics.

LR is a common and basic algorithm, which is widely used in disease risk prediction and epidemiology [39]. SVM is a discriminative classification technique, which has been widely applied in medical diagnostics and other fields, especially with small sample sets [40]. ANN, inspired by biological neural networks, has a remarkable ability to determine the meaning and rules of complicated data [41, 42]. RF, an ensemble algorithm, applies a bootstrap algorithm to extract multiple samples from the training set randomly, and trains the samples with the weak classifier (i.e. decision tree) [43]. RF’s final result is determined by the majority of votes over all decision trees, thereby improving its predictive accuracy and preventing the model from over-fitting. XGBoost is a distributed gradient boosting algorithm and has gained wide popularity and attention in machine learning competitions [44, 45]. XGBoost chooses a weak classifier to facilitate efficient optimization algorithms, adds an L2 regularization term of leaf weights to achieve lower variance, and uses the second-order Taylor series as the cost function to retain more information about the target function, thereby improving its predictive accuracy. LightGBM is a distributed and high-performance gradient lifting framework based on a decision tree algorithm designed for fast computational time, especially with very large data sets [46]. It utilizes two novel techniques: gradient-based one-side sampling and exclusive feature bundling, which respectively are used to deal with the huge number of data samples and massive amount of features [47].

All above-mentioned models were trained and tested on a partitioned 80/20 percentage split of the dataset by stratified random sampling. Simultaneously, in situations where there was imbalanced class data combined with unequal error costs, these models’ performance metrics were not representative of reasonable performances. Therefore, it was necessary to balance the dataset to get true performance values for the classifier; hence, we adjusted weights inversely proportional to class frequencies in the input data when training the machine learning models.

The parameters of these six predictive models were determined by grid search and 10-fold cross-validation in training the dataset. To be specific, we partitioned the training dataset into ten equally sized pieces, and we utilized the grid search with nine pieces to tune the parameters, while the remaining piece was used as the validation set. We repeated this process ten times. The best parameters for predictive models were obtained with the best score, which itself was obtained by averaging the process of repetition mentioned in the previous sentence. Table 2 shows the values of the parameters for each model.

**Table 2:** Summary of parameter values in each model

**Models**	**Parameters**	**Values**	**Parameters Mean**
LR	penalty	L1	penalty function
SVM	kernel	linear	kernel function
	C	5	penalty parameter of the error term
ANN	kernel initializer	uniform	kernel initializer function
	activation1	relu	activation of hidden layer
	activation2	sigmoid	activation of output layer
	optimizer	Adam	training optimization algorithm
	epochs	300	number of times shown to the network
	batch size	20	batch size
	dropout	0.0	dropout rate
RF	n estimators	695	number of iterations
	max depth	4	maximum depth of variable interactions
	max features	7	number of features for the best split
XGBoost	learning rate	0.1	learning rate
	n estimators	100	number of iterations
	eta	0.01	control of learning rate
	max depth	3	maximum depth of variable interactions
	gamma	0.6	minimum loss reduction required to make a further partition on the tree’ leaf node
	subsample	0.7	subsample ratio
	co-sample by tree	0.6	subsample ratio of columns when constructing each tree
	min child weight	2	sum of the minimum weights that leaf nodes need to observe
LightGBM	learning rate	0.1	learning rate
	n estimators	100	number of iterations
	max depth	8	maximum depth of variable interactions
	num leaves	10	number of leaves in each tree
	bagging fraction	0.7	percentage of sampling used in each iteration
	feature fraction	0.9	ratio of features to build the tree in each iteration
	min data in leaf	5	minimum number of records in a leaf
	min split gain	0.0	smallest gain of the split

### Model assessment

We calculated the AUC from receiver operating characteristic (ROC) analysis to evaluate the predictive utilities of the models, and the AUC of the six machine learning models was compared based on the DeLong method (*p*-value < 0.05 was deemed to indicate statistical significance) [48]. Meanwhile, logarithmic loss function (log-loss) was applied to quantify the accuracy of the classifier by punishing the wrong classification. Furthermore, the evaluation indicators of the confusion matrix, including accuracy, sensitivity, specificity, precision, and F1 score, were used to analyze the relationship between the actual values and the predicted values for the peak demand of CVDs admissions.

1 $$ Accuracy=\frac{TP+ TN}{TP+ TN+ FP+ FN} $$

2 $$ Sensitivity=\frac{TP}{TP+ FN} $$

3 $$ Specificity=\frac{TN}{TN= FP} $$

4 $$ \Pr ecision=\frac{TP}{TP+ FP} $$

5 $$ F1\kern0.2em score=\frac{2\ast \Pr ecision\ast \operatorname{Re} call}{\Pr ecision+\operatorname{Re} call} $$

where, TP = True Positive, FP = False Positive, TN = True Negative, FN = False Negative; $\operatorname{Re} call=\frac{TP}{TP+ FN}$

## Results

### Descriptive statistics

The statistical information of daily CVDs hospital admissions, meteorological conditions and air pollutants concentrations is summarized in Table 3. During the study period, the average of daily hospital admissions for CVDs was 208 inpatients, the minimum value was 33, and the maximum value was 476. The daily average levels of temperature, relative humidity and rainfall were 17.0 °C, 80.4% and 2.6 mm, respectively. The daily average concentrations were 60.3 μg/m^3^ for PM_2.5_, 99.3 μg/m^3^ for PM_10_, 39.0 μg/m^3^ for PM_C_, 13.9 μg/m^3^ for SO_2_, 55.0 μg/m^3^ for NO_2_, 96.0 μg/m^3^ for O_3_ and 1.1 mg/m^3^ for CO.

**Table 3:** Summary statistics of daily CVDs admissions, meteorological conditions and air pollutants concentrations in Chengdu, 2015–2017

	**Mean**	**Standard Deviation**	**Minimum**	**Median**	**Maximum**
**CVDs hospital admissions (n)**	208	90	33	206	476
**Meteorological Conditions**					
Temperature (°C)	17.0	7.2	−1.1	17.8	30.2
Relative Humidity (%)	80.4	8.8	43.0	80.8	98.3
Rainfall (mm)	2.6	8.7	0.0	0.0	122.0
**Air Pollutants Concentrations**					
PM_2.5_ (μg/m^3^)	60.3	42.4	6.1	48.4	324.5
PM_10_ (μg/m^3^)	99.3	64.7	14.3	79.8	492.5
PM_C_ (μg/m^3^)	39.0	25.8	4.8	31.6	238.2
SO_2_ (μg/m^3^)	13.9	5.8	3.9	12.7	37.9
NO_2_ (μg/m^3^)	55.0	17.3	15.7	53.0	130.4
O_3_ (μg/m^3^)	96.0	54.6	5.6	85.3	290.4
CO (mg/m^3^)	1.1	0.4	0.4	1.0	2.8

*CVDs* Cardiovascular diseases

### Evaluation and comparison of the predictive models

**Fig. 2:**
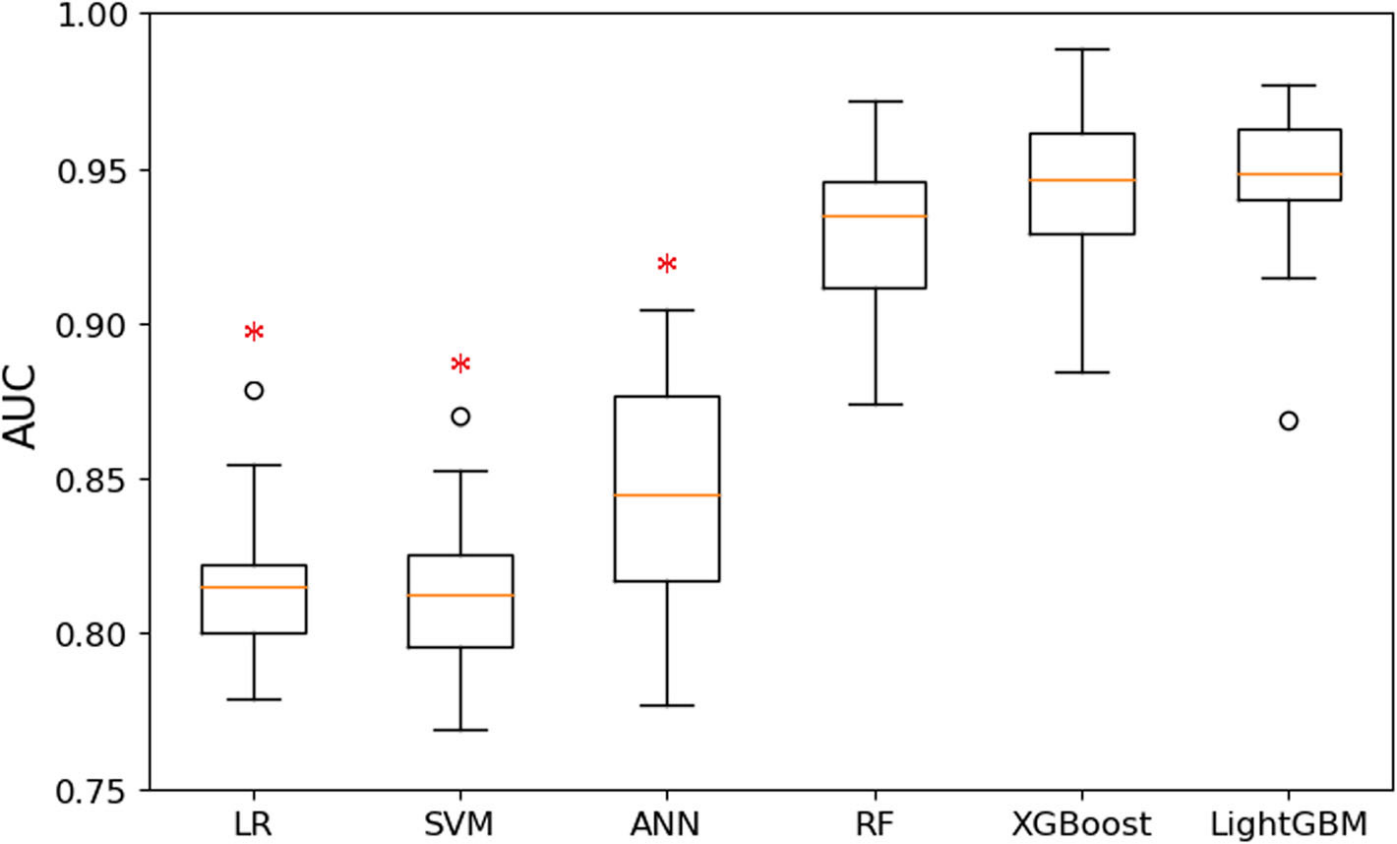
Box plot of AUC for machine learning models with 10-fold cross-validation in training dataset. °: the outliers of box plot; *: the model is significantly different from the XGBoost model. LR: logistic regression; SVM: support vector machine; ANN: artificial neural network; RF: random forest; XGBoost: extreme gradient boosting; LightGBM: light gradient boosting machine

Based on the above-mentioned features in Table 1, we constructed six machine learning models to predict the peak demand days for CVDs admissions. Using the optimal parameters for each model, the predictive models were corroborated via a validation set which was derived from the training dataset by 10-fold cross-validation. The box plot of AUC for each model with 10-fold cross-validation in training dataset is shown in Fig. 2. The AUC for LR, SVM, ANN, RF, XGBoost and LightGBM was 0.817 (95% confidence interval (CI): 0.795–0.839), 0.814 (95% CI: 0.792–0.836), 0.844 (95% CI: 0.814–0.875), 0.929 (95% CI: 0.906–0.951), 0.945 (95% CI: 0.922–0.967) and 0.9454 (95% CI: 0.921–0.967), respectively. The XGBoost model achieved the best AUC, and its performance was significantly better than LR (*p*-value < 0.001), SVM (*p*-value < 0.001) and ANN (*p*-value < 0.001), but did not differ significantly from RF (*p*-value = 0.264) and LightGBM (*p*-value = 0.933).

**Fig. 3:**
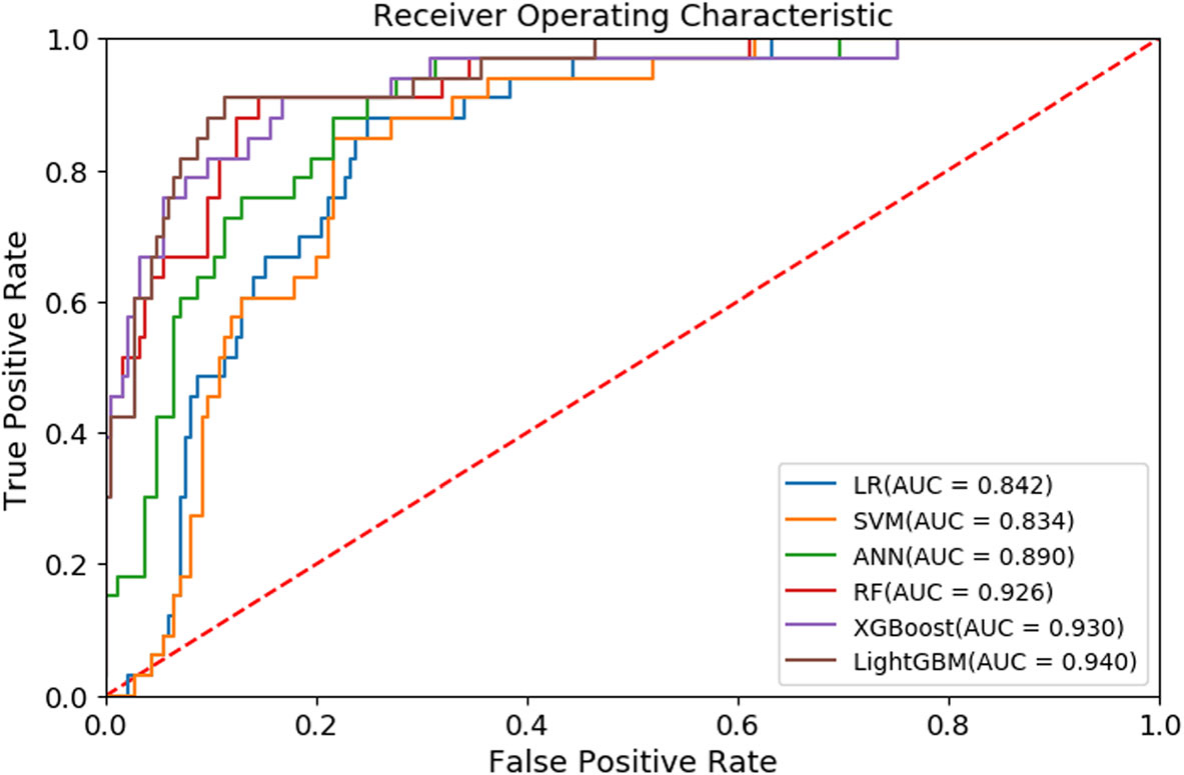
ROC curve of machine learning models in testing dataset. *LR: logistic regression; SVM: support vector machine; ANN: artificial neural network; RF: random forest; XGBoost: extreme gradient boosting; LightGBM: light gradient boosting machine*

Based on the validation result for the training dataset, we predicted the peak demand days for CVDs admissions in an independent testing dataset. The ROC curve for the predictive models in that testing dataset is shown in Fig. 3. The AUC of LR, SVM, ANN, RF, XGBoost and LightGBM was 0.842 (95% CI: 0.783–0.901), 0.834 (95% CI: 0.774–0.894), 0.890 (95% CI: 0.836–0.944), 0.926 (95% CI: 0.879–0.974), 0.930 (95% CI: 0.878–0.982) and 0.940 (95% CI: 0.900–0.980), respectively. The LightGBM model had the highest AUC value among all these predictive models, and the performance was significantly better than LR (*p*-value < 0.001), SVM (*p*-value < 0.001), ANN (*p*-value = 0.03), but did not differ significantly from RF (*p*-value = 0.222) and XGBoost (*p*-value = 0.489).

Furthermore, we used log-loss, accuracy, sensitivity, specificity, precision, and F1 score to compare the performances of these six machine learning models in the independent testing dataset (Table 4). The LightGBM model exhibited the best AUC (0.940), log-loss (0.218), accuracy (0.913), specificity (0.941), precision (0.695), and F1 score (0.725) in this testing dataset, and the RF model had the best sensitivity (0.909). Thus, the LightGBM model achieved the best performance among the six machine learning models.

**Table 4:** The evaluation indicators of machine learning models in testing dataset

**Models**	**AUC**	**log-loss**	**Accuracy**	**Sensitivity**	**Specificity**	**Precision**	**F1 score**
LR	0.842 (95% CI: 0.783–0.901)	0.513	0.766	0.848	0.751	0.378	0.523
SVM	0.834 (95% CI: 0.774–0.894)	0.344	0.748	0.879	0.724	0.362	0.513
ANN	0.890 (95% CI: 0.836–0.944)	0.296	0.858	0.333	0.951	0.551	0.415
RF	0.926 (95% CI: 0.879–0.974)	0.358	0.862	**0.909**	0.854	0.527	0.667
XGBoost	0.930 (95% CI: 0.878–0.982)	0.277	0.876	0.818	0.886	0.563	0.667
**LightGBM**^**a**^	**0.940 (95% CI: 0.900–0.980)**	**0.218**	**0.913**	0.758	**0.941**	**0.695**	**0.725**

font bold: the optimal values; ^a^the optimal model. *LR* logistic regression, *SVM* support vector machine, *ANN* artificial neural network, *RF* random forest, *XGBoost* extreme gradient boosting, *LightGBM* light gradient boosting machine

### The identification of feature importance

**Fig. 4:**
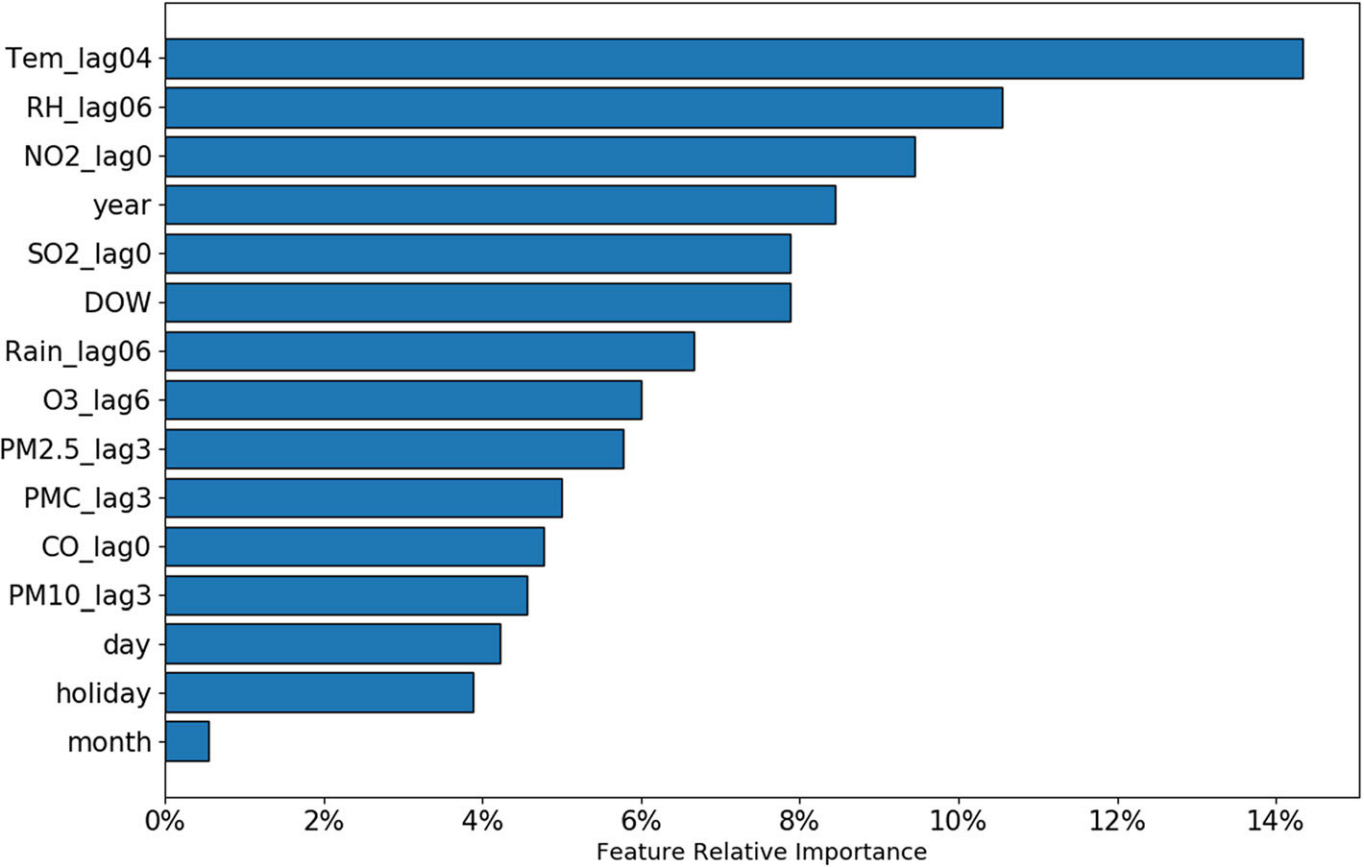
Features importance ranking based on LightGBM model

As illustrated in the above section, the LightGBM model achieved the best performance; it offers the most powerful predictors for predicting the peak demand days of CVDs admissions. The identification of feature importance based on LightGBM is shown in Fig. 4. The contribution rate of time series features, meteorological conditions and air pollutants for predicting the peak demand days of CVDs admissions was 25, 32 and 43%, respectively. Among the meteorological condition features, the top-ranked features were Tem_lag04 and RH_lag06, respectively. Similarly, the top-ranked features among the air pollutants were NO2_lag0 and SO2_lag0, respectively.

## Discussion

The six machine learning models were developed to predict the peak demand days for CVDs admissions, and as a result of our study, the optimal model has been identified. To the best of our knowledge, no studies have applied machine learning models other than ANN in the prediction of peak event of healthcare demand. This is the first study to construct and compare various machine learning models in terms of predicting the peak events of CVDs admissions using meteorological data, air quality data and hospital admissions data.

Our study found that the ensemble learning models, including LightGBM, RF and XGBoost, outperformed ANN, SVM and LR, achieved overall accuracies of > 0.86 and AUCs of > 0.92. This suggests that the ensemble learning models have better generalization capabilities compared to other models for predicting the peak demand days of CVDs admissions. The LightGBM exhibited the best performance among the ensemble learning models. Compared with ANN, SVM and LR, the AUC of LightGBM significantly improved by 5.65, 12.66 and 11.61%, respectively. Even though most predictive models have higher recall and lower precision, this could be acceptable as insufficient allocation of medical resources in peak days can lead to costly outcomes. The results of our study indicate that ensemble learning models are well suited for the prediction of peak demand for healthcare services.

The lag patterns of meteorological conditions and air pollutants have been well-documented in epidemiological studies [8, 12, 16], and suggest that the lag effects of environmental exposure have regional differences. However, to date, very few machine-learning based studies have analyzed the lag effect of environmental exposure when predicting the peak demand for healthcare services. Krishan et al. [31] applied representative lags to predictors based on the results from other studies to forecast the peak demand days of emergency department visits, but did not incorporate the actual situation of the study area. In our study, we utilized GAM to analyze the lag effect of meteorological conditions and air pollutants on CVDs admissions in our study areas. GAM is useful in the detection of early warning signals for future peak demand.

Environmental exposure, such as ambient air pollution and extreme temperatures, is an important but underappreciated risk factor contributing to the development and severity of CVDs [4]. Accumulating evidence from epidemiological studies has linked environmental exposure to increased risk of CVDs morbidity [5–12]. However, evidence of the effect of a complex mixture of environmental exposure on CVDs morbidity is still limited. Machine learning techniques provide an opportunity for developing algorithms that classify individuals with complex interaction factors. In our study, the contribution of the special ambient air pollutants and climatic characteristics of the area to the peak demand days of CVDs admissions was successfully modeled. The identification of feature importance based on the optimal model showed that among the environmental exposure features, the 4 top-ranked features were Tem_lag04, RH_lag06, NO2_lag0 and SO2_lag0, respectively, and the contribution rate of meteorological conditions and air pollutants to the prediction was 32 and 43%, respectively. These results suggest that environmental exposure is an important predictor.

Our study has several strengths. First, considering the lag effects of the complex mixture of environmental exposure and their regional differences, we utilized an over-dispersed GAM to analyze the lag effects of meteorological conditions and air pollutants on CVDs admissions, and chose the lag day with the minimum GCV value as the optimal predictor, rather than using the current day or relying on previous research, which makes our predictive models more practical. In addition, we applied six well-accepted machine learning algorithms to construct predictive models, which indicate our commitment to present a wide variety of approaches. Specially, LR represents the basic machine learning model, SVM and ANN are widely used in prediction, and RF, XGBoost and LightGBM are ensemble learning models. As discussed earlier, we found that ensemble learning models, especially the LightGBM model, have higher prediction capabilities than LR or ANN, which can benefit decision makers in finding more suitable models for the prediction of healthcare demand, especially during peak events. To the best of our knowledge, this study is the first to develop and compare various well-accepted machine learning models to predict the peak events of CVDs admissions that consider environmental exposure. Our results contribute to the limited research in this filed, as they provide useful and comprehensive information to those who seek to identify the most suitable model for decision making.

Our study also has some limitations that need to be addressed. First, we considered only two well-studied environmental exposures: meteorological conditions and ambient air pollutants, but some other environmental factors, such as exposure to the metals arsenic, cadmium and lead, also play important roles in the development and severity of CVDs [4]. Second, we just constructed the classification models to predict the peak demand days of CVDs admissions. Further study is required to forecast the number of admissions for CVDs accurately based on regression models. Third, the current model is designed for non-communicable diseases, such as CVDs, which are associated with environmental exposure, and the model might not be suitable for forecasting the peak events of infectious diseases.

## Conclusions

This study used machine learning approaches to forecast the peak demand days for CVDs admissions based on hospital admissions data, air quality data and meteorological data. The results revealed that ensemble learning models, especially the LightGBM model, can accurately predict the peak events of CVDs admissions. Meanwhile, the identification of feature importance based on LightGBM indicated that meteorological conditions and air pollutants made significant contributions to the accuracy of prediction. These findings show that machine learning approaches have potential in the prediction of the peak events of CVDs, and the predictive capacity of ensemble learning models makes them valid tools supporting decisions regarding medical resource management.

## Data Availability

The meteorological and air quality datasets analyzed during the current study are available at http://data.cma.cn/ and http://www.cnemc.cn/. Daily data of hospital admissions for CVDs are available from the Health Information Center of Sichuan Province, but restrictions are applied to these data, which were used under license for the current study, and so are not publicly available. The daily number of hospital admissions for patients with CVDs are however available from authors upon reasonable requests, and with permission of the Health Information Center of Sichuan Province, China.

## Abbreviations

ANN: Artificial neural network
ARIMA: Autoregressive integrated moving average
AUC: Area under a receiver operating characteristic curve
CO: Carbon monoxide
CVDs: Cardiovascular diseases
DOW: Day of week
GAM: Generalized additive model
GCV: Generalized Cross-Validation
LightGBM: Light gradient boosting machine
LR: Logistic regression
NO_2_: Nitrogen dioxide
O_3_: Ozone
PM_2.5_: Particulate matter with aerodynamic diameter ≤ 2.5 μm
PM_10_: Particulate matter with aerodynamic diameter ≤ 10 μm
PM_C_: Particulate matter with an aerodynamic diameter between 2.5 and 10 μm
RF: Random forest
ROC: Receiver operating characteristic
SO_2_: Sulfur dioxide
SVM: Support vector machine
XGBoost: Extreme gradient boosting
